# The Medical Streets

**Published:** 1920-08-14

**Authors:** 


### THE MEDICAL STREETS.

Among a host of disturbing innovations of the times which are affecting no less those who thought their home and professional affairs were relatively established, than those who are now engaged in the increasingly difficult process of attempting to settle down to the serious practice of medicine, there comes the vexed question of house-rent revision. But whatever the effect on the individual householder may be, there are nowadays sundry obvious reactions which have followed the raising of rents above an economic level. There is, for example, the great migration from the City, where firms resisting these profiteering impositions are discovering the landlords of Westminster and adjacent districts to be less exacting and more reasonable in their demands. Again, the infection has spread to Harley Street, where consultants and others are experiencing "difficulties" with their landlords; a considerable number of houses having been recently sold, and the tenants have been served with notices raising their rents to an almost impossible price. It is now quite customary for a charge of £300 per annum to be made for the use of a very ordinary consulting-room with the usual attendance. Experience proves that refusal to pay this sum distresses the landlord not one jot; he knows he can readily find some one who will risk the expense.

It seems quite probable, however, that the time will come when fashion will not decree that an address in the region of Cavendish Square is an essential to the professional success of a consultant. Indeed, considering the amazing congestion of this area, an overcrowding which has been steadily increasing for years past, it has been a matter of surprise that more men, both young and old, have not deliberately broken with precedent. The fashion—and it is certainly nothing whatever else—has held with extraordinary tenacity, but it may, and we think it should, decline. There is no truly reasonable explanation as to why a consultant *must* have as his environment an establishment which tends to absorb so large a proportion of his income. The truth that relatively few of them are financially able to stand the strain is evidenced to even the most casual observer by the remarkable array of brass plates upon their doors. It is true that the householder's necessity allows the young consultant at the beginning of his career to claim a Harley Street address, but even in the days before the war the price of such accommodation was held to be rising out of all proportion to the benefits conferred. How much greater is the disadvantage experienced to-day?

There is, however, one method of escape from such tyranny of fashion. There should be a realisation that the W. 1 district is not the only one in which a consultant practice can be carried on. The already established and older men will doubtless express their criticisms of this view, but we know of many instances in which the younger generation have ventured the change and have succeeded. The more of them who go further afield—and it need be really but very little further—the more rapidly will a somewhat senseless fashion change. The medical profession must institute the change, and the public, easily led but more difficult to drive, will undoubtedly sympathise with a reasonable and needed reform.

